# National Costs Associated With Methicillin-Susceptible and Methicillin-Resistant *Staphylococcus aureus* Hospitalizations in the United States, 2010–2014

**DOI:** 10.1093/cid/ciy399

**Published:** 2018-05-12

**Authors:** Eili Y Klein, Wendi Jiang, Nestor Mojica, Katie K Tseng, Ryan McNeill, Sara E Cosgrove, Trish M Perl

**Affiliations:** 1 Department of Emergency Medicine, Johns Hopkins Bloomberg School of Public Health, Baltimore, Maryland; 2 Division of Infectious Diseases, Department of Medicine, Johns Hopkins Bloomberg School of Public Health, Baltimore, Maryland; 3 Armstrong Institute for Patient Safety and Quality, Johns Hopkins University School of Medicine, Baltimore, Maryland; 4 Department of Epidemiology, Johns Hopkins Bloomberg School of Public Health, Baltimore, Maryland; 5 Center for Disease Dynamics, Economics & Policy, Washington, DC; 6 Reuters News Agency, New York; 7 City University of New York Graduate School of Journalism, New York; 8 Department of Medicine, Division of Infectious Diseases and Geographic Medicine, University of Texas Southwestern Medical Center, Dallas

**Keywords:** hospitalization costs, national inpatient sample, excess cost of resistant infections, propensity score–adjusted costs, antimicrobial resistance

## Abstract

**Background:**

Infections caused by methicillin-resistant *Staphylococcus aureus* (MRSA) have been associated with worse patient outcomes and higher costs of care than methicillin-susceptible (MSSA) infections. However, since prior studies found these differences, the healthcare landscape has changed, including widespread dissemination of community-associated strains of MRSA. We sought to provide updated estimates of the excess costs of MRSA infections.

**Methods:**

We conducted a retrospective analysis using data from the National Inpatient Sample from the Agency for Healthcare Research and Quality for the years 2010–2014. We calculated costs for hospitalizations, including MRSA- and MSSA-related septicemia and pneumonia infections, as well as MRSA- and MSSA-related infections from conditions classified elsewhere and of an unspecified site (“other infections”). Differences in the costs of hospitalization were estimated using propensity score–adjusted mortality outcomes for 2010–2014.

**Results:**

In 2014, estimated costs were highest for pneumonia and sepsis-related hospitalizations. Propensity score–adjusted costs were significantly higher for MSSA-related pneumonia ($40725 vs $38561; *P* = .045) and other hospitalizations ($15578 vs $14792; *P* < .001) than for MRSA-related hospitalizations. Similar patterns were observed from 2010 to 2013, although crude cost differences between MSSA- and MRSA-related pneumonia hospitalizations rose from 25.8% in 2010 to 31.0% in 2014. Compared with MSSA-related hospitalizations, MRSA-related hospitalizations had a higher adjusted mortality rate.

**Conclusions:**

Although MRSA infections had been previously associated with higher hospitalization costs, our results suggest that, in recent years, costs associated with MSSA-related infections have converged with and may surpass costs of similar MRSA-related hospitalizations.

## Teaser

Prior research found that costs for methicillin-resistant *Staphylococcus aureus* (MRSA) infections were greater than methicillin-susceptible (MSSA) infections. However, analysis of recent US national hospitalization data found that costs for MSSA infections are the same or greater than MRSA infections.

*Staphylococcus aureus* infections range from mild skin and soft tissue infections to serious systemic infections. Infections due to these organisms can be susceptible or resistant to methicillin. The first reports of methicillin-resistant *S. aureus* (MRSA) were published in 1961 [1, 2]. Since then, MRSA infections have reached global epidemic proportions, and MRSA is the leading cause of mortality due to antibiotic-resistant infections in the United States [3]. Recent national reports indicate that there are close to 10 MRSA-related hospitalizations per 1000 hospitalizations; this accounts for nearly 60% of all *S. aureus*–related hospitalizations [4]. For this reason, many healthcare institutions have focused on prevention of MRSA, although methicillin-susceptible *S. aureus* (MSSA) infections continue to cause a considerable number of *S. aureus* infections [4, 5].

Traditionally, MRSA infections have been associated with worse outcomes and higher costs of treatment than MSSA infections [6–13]. However, most of the studies that found these differences occurred more than a decade ago, thereby limiting their application to the current clinical environment, which in recent years has been altered by changes in the insurance landscape, the development of new diagnostics, the “save sepsis” campaign, and the emergence of community-associated MRSA and its subsequent spread into hospitals [4]. For example, heightened awareness of antimicrobial resistance, particularly MRSA, may have altered empiric prescribing practices [14]. In addition, although prior studies controlled for risk factors, such as age, exposure to invasive procedures, and comorbidities that may predispose MRSA patients to lengthier and more costly hospitalizations [15], differences amongs the cohorts and the risk factors that predispose them to MRSA infections may still remain. Newer statistical methods can improve the effectiveness of cohort matching, allowing for improved analysis of the outcomes and cost differences among patients in observational studies using data captured from administrative databases [15].

An understanding of the magnitude of, and trends in, the excess costs of resistant infections at a national level is necessary for developing rational responses to the growing public health crisis of antibiotic resistance. Although studies have examined the difference in costs between MRSA and MSSA infections at the hospital level, to the best of our knowledge, no study has attempted to understand national-level cost differences in the United States. To improve societal estimates of and to examine changes in the excess costs of MRSA infections on overall hospitalization costs in the United States, we used national hospitalization data to estimate the overall costs of treating patients diagnosed with *S. aureus* infections and the difference in treatment costs between patients with MRSA infections and those with MSSA infections over a 5-year period. We used propensity-score analysis to minimize biases associated with comparisons of nonrandomized populations.

## METHODS

We calculated costs associated with MRSA- and MSSA-related hospitalizations from 2010 to 2014 using the National Inpatient Sample (NIS) from the Healthcare Cost and Utilization Project of the Agency for Health Research and Quality. The NIS approximates a 20% stratified sample of all discharges from community hospitals in the United States each year [16]. Each record in the database is weighted to produce national estimates of hospitalization and provides data on patient demographics, diagnoses, mortality, and charges related to hospital care. The NIS data are de-identified public-use files that do not require institutional review board approval for use in research.

Diagnoses in the NIS from 2010 through 2014 were coded using the *International Classification of Diseases, Ninth Revision, Clinical Modification* (ICD-9). Up to 25 diagnoses were available for each patient record in 2010–2013 and up to 30 diagnoses were available in 2014. Similar to prior analyses [4, 5], an MSSA or MRSA hospitalization was defined as an encounter using ICD-9 diagnoses. All records containing an MSSA or MRSA infection code were included: *S. aureus* septicemia (038.11, 038.12), *S. aureus* pneumonia (482.41, 482.42), and “other” *S. aureus* infections in conditions classified elsewhere and of an unspecified site (041.11, 041.12). Records with multiple *S. aureus* diagnoses were only counted once, with preference given to septicemia, followed by pneumonia and then other. Visits with both an MRSA and MSSA infection code were considered MRSA-related hospitalizations.

Hospitalization costs are presented in 2014 US dollars and were calculated by multiplying patient hospital charges by the cost-to-charge ratio provided in the NIS [17]. The NIS uses cost files reported annually by hospitals to the Centers for Medicare and Medicaid Services to generate the all-payer inpatient cost-to-charge ratio (APICC), which is the inpatient costs divided by the inpatient charges. The APICC populates the NIS Cost-to-Charge Ratio files, except when hospital-specific APICC data are not available, whereby the group average cost-to-charge ratio, which weights hospitals against their peer group based on bed count, is used instead.

To reduce potential selection bias due to differences between patients with MRSA and those with MSSA and to account for risk factors that may predispose MRSA patients to lengthier and more costly hospitalizations prior to infection onset [15], we used propensity-score matching to estimate the cost difference between MRSA- and MSSA-related hospitalizations, taking into account the complex NIS survey design [18]. Clinical decisions on antibiotic therapy, particularly empirical therapy, which can greatly affect a patient’s hospitalization course, are guided by patient pathology and conditional patient-related factors. The conditional patient factors included in the estimation of the propensity scores were age, race, hospital region, Charlson score [19], All Patients Refined Diagnosis Related Groups severity and risk mortality scores, primary diagnosis, the number of procedures performed and diagnoses listed on the record, and length of stay (LOS). Crude and propensity score–adjusted mean total costs and likelihood of mortality were calculated in total and separately for septicemia, pneumonia, and other *S. aureus*–related hospitalizations. The adjusted Wald test was used to compare differences in propensity score–adjusted costs between MSSA and MRSA infections. To compare differences in the mortality rate between MSSA and MRSA related-hospitalizations, the Charlson score was collapsed for values >10 due to collinearity. Analyses were performed using Stata 14.1 (StataCorp, College Station, TX) and accounted for the complex sampling design of the NIS.

Because mortality differences and long hospitals stays could bias results, we included an analysis of cost differences between MRSA- and MSSA-related hospitalizations that excluded patients that died in the hospital, and then we further restricted this population to patients that had an LOS <10 days (the approximate mean LOS). Because skin and soft tissue infections (SSTIs) primarily drive the dynamics of other *S. aureus* hospitalizations [4, 5], we also conducted an analysis of cost differences among MRSA- and MSSA-related hospitalizations with and without SSTIs. Skin and soft tissue infections were classified as any “other” *S. aureus* hospitalization (based on the aforementioned definition) that included an SSTI ICD-9 code defined in May et al [20]. The included codes were carbuncle and furuncle (680.xx), cellulitis and abscess of finger and toe (681.xx), impetigo (684.xx), other cellulitis and abscess (682.xx), other local infections of skin and subcutaneous tissue (686.xx), inflammatory disease of breast (611.0), other specified diseases of hair and hair follicles (704.8), and erysipelas (35).

## RESULTS

Overall, there were no large differences in demographics, LOS, Charlson score, or All Patients Refined Diagnosis Related Groups severity and risk mortality scores between patients with MRSA- related hospitalizations and those with MSSA-related hospitalizations in any year (see Table 1 for 2014 data and Supplementary Table 1 for 2010–2013 data). In unadjusted descriptive analysis, patients with MRSA pneumonia-related hospitalizations had a higher median age and Charlson score, a shorter LOS, and a greater likelihood of having *S. aureus* infection as their primary diagnosis, compared with those with MSSA pneumonia-related hospitalization. Unadjusted mortality rates were higher for MRSA septicemia- and other *S. aureus*–related hospitalizations than for similar MSSA septicemia- and other *S. aureus*–related hospitalizations, whereas MSSA pneumonia-related hospitalizations had a higher unadjusted mortality rate than similar MRSA pneumonia-related hospitalizations. These differences were consistent across years with the exception of patients with MRSA pneumonia-related hospitalizations in 2011, who had the same LOS as patients with MSSA pneumonia-related hospitalizations. After propensity matching, patient demographics were similar between the 2 groups (Supplementary Table 2). Mortality rates for MRSA-related septicemia and other hospitalizations were significantly higher than mortality rates for MSSA-related hospitalizations (septicemia, *P* = .004; other, *P* < .001) but were similar for pneumonia (*P* = .73). Differences in mortality were consistent for years 2010–2013, with the exception of mortality rates in 2011 and 2012, which were significantly higher (*P* = .02) for MRSA-related pneumonia hospitalizations than for similar MSSA-related hospitalizations.

**Table 1.** Patient Demographics for *Staphylococcus aureus*–Related Hospitalizations, 2014

	Overall		Septicemia^a^		Pneumonia^b^		Unspecified^c^	
	MRSA	MSSA	MRSA	MSSA	MRSA	MSSA	MRSA	MSSA
**Total No.**	358140	257930	54255	57065	48780	25020	255105	175845
Median age, y (IQR)	59 (43–74)	57 (42–70)	63 (50–75)	61 (48–72)	67 (54–79)	60 (45–72)	57 (40–72)	55 (40–68)
Race								
White	245615 (71.7)	171735 (70.7)	35165 (68.0)	37200 (69.7)	35785 (76.7)	17130 (73.3)	174665 (71.5)	117405 (70.7)
Black	49495 (14.4)	31370 (12.9)	9435 (18.3)	7895 (14.8)	5705 (12.2)	2640 (11.3)	34355 (14.1)	20835 (12.5)
Hispanic	30925 (9.0)	25710 (10.6)	4535 (8.8)	5205 (9.8)	2945 (6.3)	2125 (9.1)	23445 (9.6)	18380 (11.1)
Other	16430 (4.8)	14125 (5.8)	2540 (4.9)	3110 (5.8)	2205 (4.7)	1490 (6.4)	11685 (4.8)	9525 (5.7)
Sex								
Male	194750 (54.4)	152105 (59.0)	31175 (57.5)	34250 (60.0)	25880 (53.1)	14845 (59.4)	137695 (54.0)	103010 (58.6)
Female	163305 (45.6)	105775 (41.0)	23065 (42.5)	22810 (40.0)	22895 (46.9)	10165 (40.6)	117345 (46.0)	72800 (41.4)
Region								
Northeast	57595 (16.1)	48920 (19.0)	8800 (16.2)	11230 (19.7)	7190 (14.7)	4125 (16.5)	41605 (16.3)	33565 (19.1)
Midwest	76900 (21.5)	59255 (23.0)	11255 (20.7)	12745 (22.3)	10470 (21.5)	5440 (21.7)	55175 (21.6)	41070 (23.4)
South	158925 (44.4)	93550 (36.3)	23275 (42.9)	19575 (34.3)	22425 (46.0)	9645 (38.6)	113225 (44.4)	64330 (36.6)
West	64720 (18.1)	56205 (21.8)	10925 (20.1)	13515 (23.7)	8695 (17.8)	5810 (23.2)	45100 (17.7)	36880 (21.0)
Primary diagnosis of *Staphylococcus aureus* infection	48485 (13.5)	38435 (14.9)	34410 (63.4)	33840 (59.3)	13725 (28.1)	4530 (18.1)	350 (0.1)	65 (0.0)
Median LOS, d (IQR)	6 (4–11)	7 (4–12)	10 (6–17)	9 (6–15)	10 (6–18)	12 (7–20)	5 (3–9)	6 (3–9)
Mean Charlson Score (SD)	2.30 (2.26)	2.21 (2.27)	2.98 (2.38)	2.92 (2.40)	2.82 (2.26)	2.49 (2.33)	2.06 (2.18)	1.94 (2.15)
Severity (SD)	2.75 (0.98)	2.80 (0.95)	3.48 (0.67)	3.43 (0.67)	3.57 (0.67)	3.70 (0.59)	2.43 (0.91)	2.47 (0.89)
Risk mortality (SD)	2.35 (1.10)	2.35 (1.11)	3.28 (0.86)	3.20 (0.86)	3.28 (0.83)	3.39 (0.80)	1.97 (0.96)	1.92 (0.95)
Mean no. of procedures performed (SD)	2.55 (2.99)	3.01 (3.18)	3.93 (3.58)	3.92 (3.53)	3.70 (3.82)	4.90 (4.21)	2.03 (2.47)	2.45 (2.68)
Mean no. of diagnoses (SD)	15.45 (7.14)	15.30 (6.93)	19.02 (6.23)	18.50 (6.13)	18.54 (6.16)	18.35 (6.24)	14.10 (7.04)	13.82 (6.78)
Died	16485 (4.6)	11305 (4.4)	6685 (12.3)	5955 (10.4)	5760 (11.8)	3030 (12.1)	4040 (1.6)	2320 (1.3)

Data are no. (%) unless otherwise indicated.

Abbreviations: IQR, interquartile range; LOS, length of stay; MRSA, methicillin-resistant *Staphylococcus aureus*; MSSA, methicillin-susceptible *Staphylococcus aureus*; SD, standard deviation.

^a^ *International Classification of Diseases, Ninth Revision, Clinical Modification* (ICD-9) codes 038.11 and 038.12.

^b^ ICD-9 codes 482.41 and 482.42.

^c^ ICD-9 codes 041.11 and 041.12.

Hospitalization costs in 2014 were highest for pneumonia-related hospitalizations, followed by septicemia-related hospitalizations, and other *S. aureus*–related hospitalizations (Table 2). Crude costs for MSSA-related hospitalizations were greater than MRSA-related costs for both pneumonia and other *S. aureus*–related infections, although costs were approximately the same for septicemia-related hospitalizations. Propensity score–adjusted costs for MSSA pneumonia- and other *S.aureus*–related hospitalizations were 5.5% ($40725 vs $38561; *P* = .045) and 5.2% ($15578 vs $14792; *P* < .001) higher than for MRSA-related hospitalizations, respectively. Methicillin-susceptible *S. aureus*–related septicemia hospitalization costs were not significantly different from MRSA-related hospitalization costs ($34526 vs $34175; *P* = .69). However, among pneumonia-related hospitalizations, patients with MRSA infections had a higher rate of mortality than patients with MSSA infections (*P* < .001).

**Table 2.** Crude and Propensity Score–Adjusted Difference in Costs Between Methicillin-resistant *Staphylococcus aureus*- and Methicillin-susceptible *Staphylococcus aureus*-Related Hospitalizations, 2014

	Crude costs, $^a^		Propensity score–adjusted costs, $^a^		
	MRSA	MSSA	MRSA	MSSA	*P* Value
Overall (n = 616070)					
Septicemia^a^	35408 (34007–36808)	34628 (33154–36102)	34526 (32918–36134)	34175 (32347–36002)	.69
Pneumonia^b^	39589 (38048–41131)	54132 (51632–56632)	38561 (36811–40725)	40725 (38717–42733)	.045
Unspecified^c^	15948 (15516–16380)	18977 (18262–19692)	14792 (14383–15201)	15578 (15079–16077)	<.001
Hospitalizations with no in-hospital mortality (n = 587850)					
Septicemia^a^	33564 (32224–34904)	32440 (31157–33723)	32618 (31072–34164)	32170 (30585–33754)	.589
Pneumonia^b^	37737 (36162–39311)	52678 (50060–55296)	36771 (34989–38553)	39041 (37015–41066)	.047
Unspecified^c^	15637 (15218–16057)	18545 (17879–19212)	14529 (14132–14926)	15314 (14828–15799)	<.001
Hospitalizations with no in-hospital mortality (LOS < 10 days) (n = 407040)					
Septicemia^a^	13269 (12999–13538)	14130 (13845–14416)	13204 (12930–13479)	13886 (13594–14177)	<.001
Pneumonia^b^	12962 (12661–13264)	15483 (14871–16096)	12870 (12565–13175)	13763 (13266–14259)	.001
Unspecified^c^	9131 (9009–9252)	10248 (10088–10408)	9106 (8981–9230)	9576 (9432–9720)	<.001

Abbreviations: LOS, length of stay; MRSA, methicillin-resistant *Staphylococcus aureus*; MSSA, methicillin-susceptible *Staphylococcus aureus*.

^a^Mean total cost (95% confidence interval).

^b^ *International Classification of Diseases, Ninth Revision, Clinical Modification* (ICD-9) codes 038.11 and 038.12.

^c^ICD-9 codes 482.41 and 482.42.

^d^ICD-9 codes 041.11 and 041.12.

Results after excluding patients that died in the hospital or had an LOS >10 days were generally consistent with the overall analyses, with MSSA pneumonia- and other *S. aureus*–related hospitalizations being more costly than similar MRSA-related hospitalizations (Table 2). However, although propensity score–adjusted costs were similar for septicemia-related hospitalizations with no mortality ($32170 vs $32618; *P* = .59), MSSA septicemia-related hospitalizations were more costly after restricting the analysis to hospitalizations with an LOS <10 days ($13886 vs 13204; *P* < .001).

**Figure 1.**
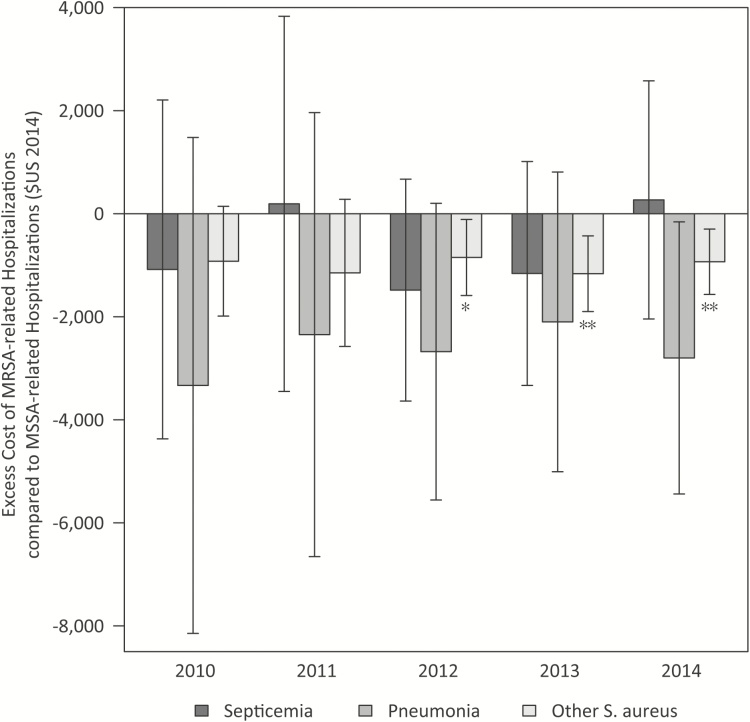
Excess cost of methicillin-resistant *Staphylococcus aureus* (MRSA) compared with methicillin-susceptible *S. aureus* (MSSA) hospitalizations, 2010–2014. The excess cost of MRSA-related hospitalizations compared wth MSSA-related hospitalizations was measured as the mean cost of MRSA-related hospitalizations minus the mean cost of MSSA-related hospitalizations. The arrows indicate the 95% confidence interval of the difference in the means. The negative values indicate that MRSA-related hospitalizations were, on average, less costly than similar MSSA-related hospitalizations. The stars indicate the *P*-value of whether the mean was significantly different from 0 at *P* < .05 (*) or *P* < .01 (**).

The magnitude of the difference in cost in each diagnosis group was similar for the years 2010–2013. The cost difference, however, was not significant in all years for all diagnosis groups (Figure 1 and Supplementary Table 3). In 2010, although MSSA-related hospitalizations were estimated to be more costly than MRSA-related hospitalizations for all diagnoses, the confidence intervals for these estimates all crossed zero. However, by 2014, the costs of MSSA other *S. aureus*–related hospitalizations was significantly greater (Figure 1), although no difference remained between MRSA and MSSA pneumonia- and septicemia-related hospitalization costs. One effect of propensity-score matching was to mitigate the magnitude of the crude cost differences, which for pneumonia-related hospitalizations were tens of thousands of dollars more costly for MSSA-related infections than for MRSA-related infections.

Skin and soft tissue infections accounted for approximately half (51.4%) of all other *S. aureus*–related hospitalizations and were far less costly than non-SSTI–related hospitalizations (Supplementary Table 4). Similar to the other analyses, costs for MSSA-related hospitalizations were greater than for MRSA related-hospitalizations for both SSTI- and non-SSTI–related hospitalizations. After propensity-score adjustments, costs for MSSA-related hospitalizations were significantly greater than the costs for MRSA-related hsopitalizations for non-SSTIs (*P* = .002) but were not statistically different for SSTIs (*P* = .17).

## DISCUSSION

Contrary to historical studies at the hospital level [6–13], we found that, nationally, MRSA-related hospitalization costs were approximately the same as or less costly than MSSA-related infections. Although MRSA infections have been associated with increased hospitalization costs [6–13], our results suggest that after adjusting for background characteristics, costs associated with MSSA-related infections have converged and may even surpass costs of similar MRSA-related hospitalizations in some contexts. From 2010 to 2014, the costs associated with MSSA pneumonia- and other *S. aureus*–related hospitalizations were higher than the costs for similar MRSA-related hospitalizations, although the costs for septicemia-related hospitalizations were similar.

There are a number of potential reasons for these cost differences based on prior studies. One reason may be the increased incidence of community-associated MRSA infections, which predominantly cause noninvasive SSTIs [4, 5] that are generally more susceptible to second-line drugs, which may have lowered the treatment costs associated with MRSA, particulary for the other infections cohort. For more invasive infections, MSSA patients may have been treated with antistaphylococcal penicillins, which are more expensive than vancomycin. However, further research is needed to test whether differences in medication costs for treating MRSA and MSSA infections may have led to overall differences in hospitalization costs because the data lacked information on antimicrobial therapy. A second potential reason for the difference from prior studies may be that MSSA infections were more severe [21]. Alternatively, less-invasive MSSA infections may not be diagnosed or coded correctly, leaving only more costly MSSA infections in the record. This may be why the mean number of procedures for MSSA-related hospitalizations was higher than that for MRSA-related hospitalizations.

A third potential reason contributing to the convergence of costs of MSSA and MRSA infections could be the increase in empiric use of vancomycin. The increase in vancomycin could have led to earlier optimal therapy for patients with MRSA, thus resulting in improved outcomes and reduced costs in these patients. However, increased empiric vancomycin therapy may also have led to a concomitant decrease in use of antistaphylococcal penicillins and cefazolin as empiric or definitive therapy for patients who ultimately grow MSSA, and patients with MSSA treated with vancomycin rather than beta-lactam agents have been shown to have worse outcomes [22]. Although the NIS does not include medications administered, future investigations should evaluate whether empiric use of vancomycin leads to poorer outcomes and higher costs for MSSA patients. Finally, heightened awareness of MRSA may have increased hospital surveillance for MRSA. This, in turn, may have increased the probability of diagnosing less invasive MRSA infections or associating nontarget microbiology results, such as nasal swabs, with other diagnoses, such as pneumonia, in the coding process. A shift in coding could potentially explain some of the differences in costs and should be investigated further.

Our results regarding higher mortality for those with MRSA infections are in line with the findings of most prior studies [23]. Mortality results were based on propensity-matched cohorts, which adjusts for comorbidities and severity of illness, suggesting that resistant infections may be a determinant of mortality. However, because of limitations of the dataset, we were not able to fully adjust for all confounding factors that drive differences between cohorts, such as source of infection, whether source control was obtained, therapy provided, timing of infection onset, lab costs, and specialist consultations. This may explain why our results differed from a recent propensity-score analysis study that found no difference in the mortality rate between patients with MRSA and patients with MSSA infections [15].

Although our results are national in scope, our findings are subject to some of the inherent limitations of using large hospitalization databases. Results are based on diagnostic billing codes, which, although widely used, may reflect a bias in reporting and billing for MRSA infections [24, 25]. Although we used cost-to-charge ratios to approximate costs for each hospitalization and to help account for variability in charges across hospitals, our findings may not reflect true costs related to these infections. For example, we were unable to separate hospitalization costs for *S. aureus* infections from the cost of treatment of concurrent conditions, although there is no reason to assume differences in the costs of comorbidities between cohorts. Lastly, although we attempted to minimize bias between MRSA patients and MSSA patients through propensity-score matching, it was not possible to match for all potential confounders. For example, MRSA patients were slightly more likely to be transferred to a different acute care hospital or another type of facility. Although the results were qualitatively the same when restricted to patients that were discharged home, it suggests that patients with an MRSA infection may be treated more cautiously in a manner that is not readily identifiable in administrative data. However, our results are similar to those from other studies comparing MSSA and MRSA bloodstream infections, which found no difference in hospitalization costs after propensity-score adjustments [15] and no increase in LOS, cost, or hazard for MRSA bloodstream infections relative to similar MSSA infections [14].

Quantifying the difference in costs and outcomes between patients infected with resistant organisms and those infected with susceptible organisms is important for prioritizing investments in antimicrobial stewardship, diagnostics, and clinical operations. However, direct observational cohorts may not be the most effective means of comparison because there may be confounders that generate differences between cohorts: patients who acquire MRSA infections may have risk factors that predispose them for resistant infections. Adjusting for differences in underlying patient severity can help ameliorate these types of issues and provide a better understanding of the economic impact of antibiotic resistance. Further research comparing outcomes and costs between resistant and susceptible pathogens should use these or similar methods and also attempt to determine the most salient confounders and more clearly delineate the relationship among resistance phenotypes, empiric therapy, and clinical outcomes.

## Supplementary Data

Supplementary materials are available at *Clinical Infectious Diseases* online. Consisting of data provided by the authors to benefit the reader, the posted materials are not copyedited and are the sole responsibility of the authors, so questions or comments should be addressed to the corresponding author.

## Supplementary Material

Supplementary Tables

## Notes

***Financial support.*** This work was supported by the Bill & Melinda Gates Foundation (OPP1112355) and the Centers for Disease Control and Prevention (16IPA1609426 and 16IPA160 to W. J. and N. M.).

***Potential conflicts of interest.*** S. E. C. reports personal fees from Theravance and Novartis, outside the submitted work. All other authors report no potential conflicts. All authors have submitted the ICMJE Form for Disclosure of Potential Conflicts of Interest. Conflicts that the editors consider relevant to the content of the manuscript have been disclosed.
