# Supplementary material for: National Costs Associated With Methicillin-Susceptible and Methicillin-Resistant Staphylococcus aureus Hospitalizations in the United States, 2010–2014
Source: Clin Infect Dis. 2018 May 12;68(1):22–8. doi: 10.1093/cid/ciy399 (PMC6293004; doi:10.1093/cid/ciy399)
Supplement: Supplementary Tables [file ciy399_suppl_supplementary_tables.docx]

| **Supplementary Table 1. Patient demographics for *Staphylococcus aureus*-related hospitalizations, 2010-2013** | | | | | | | | | |
| --- | --- | --- | --- | --- | --- | --- | --- | --- | --- |
|  | Overall in 2010 | | Overall in 2011 | | Overall in 2012 | | Overall in 2013 | |  |
|  | MRSA | MSSA | MRSA | MSSA | MRSA | MSSA | MRSA | MSSA |  |
|  | n (%)^a^ | n (%)^a^ | n (%)^a^ | n (%)^a^ | n (%)^a^ | n (%)^a^ | n (%)^a^ | n (%)^a^ |  |
| **Total, N** | 422,306 | 238,836 | 427,007 | 249,703 | 399,710 | 247,835 | 380,085 | 252,085 |  |
| Median Age (IQR) | 58 (41-74) | 55 (40-70) | 59 (43-75) | 57 (42-71) | 59 (42-74) | 57 (42-70) | 59 (43-74) | 57 (42-70) |  |
| Race |  |  |  |  |  |  |  |  |  |
| White | 266,555 (70.0) | 147,067 (68.8) | 276,527 (71.3) | 159,613 (70.6) | 272,080 (71.4) | 164,540 (70.6) | 259,055 (71.9) | 165,985 (70.2) |  |
| Black | 62,810 (16.5) | 32,575 (15.2) | 60,017 (15.5) | 31,131 (13.8) | 56,995 (15.0) | 29,935 (12.8) | 52,965 (14.7) | 30,790 (13.0) |  |
| Hispanic | 33,934 (8.9) | 22,672 (10.6) | 35,161 (9.1) | 23,001 (10.2) | 33,205 (8.7) | 24,020 (10.3) | 32,425 (9.0) | 26,270 (11.1) |  |
| Other | 17,715 (4.7) | 11,538 (5.4) | 16,141 (4.2) | 12,336 (5.5) | 18,840 (4.9) | 14,700 (6.3) | 16,100 (4.5) | 13,525 (5.7) |  |
| Gender |  |  |  |  |  |  |  |  |  |
| Male | 225,925 (53.5) | 138,860 (58.2) | 227,563 (53.3) | 144,247 (57.8) | 215,730 (54.0) | 144,560 (58.3) | 205,815 (54.2) | 148,155 (58.8) |  |
| Female | 196,082 (46.5) | 99,806 (41.8) | 199,178 (46.7) | 105,365 (42.2) | 183,960 (46.0) | 103,270 (41.7) | 174,225 (45.8) | 103,920 (41.2) |  |
| Region |  |  |  |  |  |  |  |  |  |
| Northeast | 63,918 (15.1) | 44,392 (18.6) | 69,946 (16.4) | 48,812 (19.6) | 65,130 (16.3) | 47,360 (19.1) | 61,420 (16.2) | 47,900 (19) |  |
| Midwest | 89,776 (21.3) | 53,247 (22.3) | 93,153 (21.8) | 56,788 (22.7) | 85,955 (21.5) | 56,385 (22.8) | 81,975 (21.6) | 57,680 (22.9) |  |
| South | 192,209 (45.5) | 90,197 (37.8) | 187,498 (43.9) | 92,594 (37.1) | 177,465 (44.4) | 90,655 (36.6) | 169,675 (44.6) | 92,320 (36.6) |  |
| West | 76,403 (18.1) | 51,000 (21.4) | 76,410 (17.9) | 51,509 (20.6) | 71,160 (17.8) | 53,435 (21.6) | 67,015 (17.6) | 54,185 (21.5) |  |
| Primary diagnosis of *Staphylococcus aureus* infection | 47,856 (11.3) | 27,295 (11.4) | 54,284 (12.7) | 32,421 (13.0) | 49,050 (12.3) | 32,425 (13.1) | 49,725 (13.1) | 35,695 (14.2) |  |
| Median LOS (IQR) | 6 (4-11) | 6 (4-11) | 6 (4-12) | 6 (4-11) | 6 (4-11) | 6 (4-11) | 6 (4-11) | 6 (4-11) |  |
| Mean Charlson Score (SD) | 2.05 (2.18) | 1.95 (2.18) | 2.19 (2.21) | 2.13 (2.23) | 2.18 (2.20) | 2.12 (2.21) | 2.22 (2.23) | 2.15 (2.22) |  |
| Severity (SD) | 2.76 (0.99) | 2.81 (0.96) | 2.83 (0.97) | 2.87 (0.93) | 2.65 (1.00) | 2.71 (0.97) | 2.70 (0.99) | 2.75 (0.96) |  |
| Risk Mortality (SD) | 2.26 (1.11) | 2.25 (1.12) | 2.33 (1.11) | 2.32 (1.11) | 2.26 (1.09) | 2.26 (1.09) | 2.31 (1.10) | 2.30 (1.10) |  |
| Mean number of procedures performed (SD) | 2.45 (2.87) | 2.85 (3.05) | 2.45 (2.83) | 2.89 (3.05) | 2.46 (2.84) | 2.93 (3.11) | 2.50 (2.87) | 2.96 (3.12) |  |
| Mean number of diagnoses (SD) | 12.58 (6.35) | 12.29 (6.09) | 13.62 (6.53) | 13.59 (6.31) | 13.97 (6.57) | 13.92 (6.38) | 14.43 (6.57) | 14.37 (6.38) |  |
| Died | 19,533 (4.6) | 10,243 (4.3) | 20,002 (4.7) | 10,896 (4.4) | 18,770 (4.7) | 10,425 (4.2) | 17,795 (4.7) | 10,415 (4.1) |  |
| Note. IQR = interquartile range; LOS = length of stay; MSSA = methicillin-susceptible *Staphylococcus aureus*; MRSA = methicillin-resistant *S. aureus*; SD = standard deviation.  ^a^ Unless otherwise indicated. | | | | | | | | | |

| **Supplementary Table 2. Patient demographics for propensity-adjusted *Staphylococcus aureus*-related hospitalizations, 2014** | | | | | | | |
| --- | --- | --- | --- | --- | --- | --- | --- |
|  | Septicemia^a^ | | Pneumonia^b^ | | Other^c^ | |  |
|  | MRSA | MSSA | MRSA | MSSA | MRSA | MSSA |  |
|  | n (%)^d^ | n (%)^d^ | n (%)^d^ | n (%)^d^ | n (%)^d^ | n (%)^d^ |  |
| **Total, N** | 44,975 | 44,993 | 40,315 | 40,350 | 231,190 | 231,104 |  |
| Median Age (IQR) | 63 (50-76) | 61 (48-73) | 67 (55-79) | 63 (50-75) | 56 (40-72) | 56 (41-70) |  |
| Race |  |  |  |  |  |  |  |
| White | 30,815 (68.5) | 30,880 (68.6) | 31,050 (77.0) | 31,000 (76.8) | 165,875 (71.8) | 165,355 (71.6) |  |
| Black | 8,025 (17.8) | 8,003 (17.8) | 4,830 (12.0) | 4,920 (12.2) | 32,080 (13.9) | 32,453 (14.0) |  |
| Hispanic | 3,915 (8.7) | 3,945 (8.8) | 2,525 (6.3) | 2,516 (6.2) | 22,180 (9.6) | 22,155 (9.6) |  |
| Other | 2,220 (4.9) | 2,165 (4.8) | 1,910 (4.7) | 1,913 (4.7) | 11,055 (4.8) | 11,141 (4.8) |  |
| Gender |  |  |  |  |  |  |  |
| Male | 25,995 (57.8) | 26,020 (57.8) | 21,225 (52.7) | 21,278 (52.7) | 124,505 (53.9) | 124,291 (53.8) |  |
| Female | 18,980 (42.2) | 18,973 (42.2) | 19,090 (47.4) | 19,072 (4.73) | 106,685 (46.2) | 106,813 (46.2) |  |
| Region |  |  |  |  |  |  |  |
| Northeast | 7,575 (16.8) | 7,511 (16.7) | 6,160 (15.3) | 6,216 (15.4) | 38,415 (16.6) | 38,435 (16.6) |  |
| Midwest | 8,370 (18.6) | 8,415 (18.7) | 7,940 (19.7) | 7,703 (19.1) | 46,420 (20.1) | 46,494 (20.1) |  |
| South | 19,760 (43.9) | 19,752 (43.9) | 18,955 (47.0) | 19,071 (47.3) | 104,530 (45.2) | 104,432 (45.2) |  |
| West | 9,270 (20.6) | 9,314 (20.7) | 7,260 (18.0) | 7,360 (18.2) | 41,825 (18.1) | 41,743 (18.1) |  |
| Primary diagnosis of *Staphylococcus aureus* infection | 28,715 (63.9) | 28,614 (63.6) | 12,065 (29.9) | 12,310 (30.5) | 310 (0.1) | 293 (0.1) |  |
| Median LOS (IQR) | 8 (5-13) | 8 (5-13) | 9 (6-14) | 9 (6-14) | 5 (3-8) | 5 (3-8) |  |
| Mean Charlson Score (SD) | 2.96 (2.38) | 2.97 (2.39) | 2.80 (2.25) | 2.80 (2.25) | 2.01 (2.16) | 2.02 (2.17) |  |
| Severity (SD) | 3.45 (0.68) | 3.45 (0.68) | 3.54 (0.68) | 3.53 (0.69) | 2.40 (0.90) | 2.40 (0.91) |  |
| Risk Mortality (SD) | 3.24 (0.88) | 3.24 (0.88) | 3.24 (0.84) | 3.23 (0.85) | 1.94 (0.95) | 1.95 (0.95) |  |
| Mean number of procedures performed (SD) | 3.74 (3.56) | 3.74 (3.52) | 3.48 (3.81) | 3.49 (3.70) | 1.93 (2.37) | 1.96 (2.19) |  |
| Mean number of diagnoses (SD) | 18.84 (6.15) | 18.84 (6.10) | 18.31 (6.14) | 18.28 (6.16) | 13.84 (6.97) | 13.88 (6.87) |  |
| Died | 5,710 (12.7) | 5,077 (11.3) | 4,500 (11.2) | 4,105 (10.2) | 3,445 (1.5) | 2,834 (1.2) |  |
| Note. IQR = interquartile range; LOS = length of stay; MSSA = methicillin-susceptible *Staphylococcus aureus*; MRSA = methicillin-resistant *S. aureus*; SD = standard deviation.  ^a^ ICD-9-CM: 038.11 and 038.12.  ^b^ ICD-9-CM: 482.41 and 482.42.  ^c^ ICD-9-CM: 041.11 and 041.12.  ^d^ Unless otherwise indicated. | | | | | | | |

| **Supplementary Table 3. Crude and propensity-Adjusted difference in costs between MRSA- and MSSA-related hospitalizations, 2010-2013** | | | | | |  |  |  |
| --- | --- | --- | --- | --- | --- | --- | --- | --- |
|  | Crude Costs ($)^d^ | | Propensity Score Costs ($)^d^ | | | |  |  |
|  | MRSA | MSSA | MRSA | MSSA | *p*-value | | |  |
| **Overall in 2010 (n = 661,142)** | | |  |  |  | | | |
| Septicemia^a^ | 36,309 (34,212-38,406) | 34,715 (32,916-36,513) | 35,936 (33,432-38,440) | 36,908 (34,660-39,157) | 0.324 | | |  |
| Pneumonia^b^ | 41,025 (38,367-43,682) | 53,179 (49,152-57,206) | 40,495 (37,371-43,618) | 43,398 (39,327-47,468) | 0.071 | | |  |
| Unspecified^c^ | 15,382 (14,634-16,131) | 17,590 (16,631-18,549) | 14,480 (13,737-15,223) | 15,358 (14,546-16,170) | <0.001 | | |  |
| **Overall in 2011 (n = 676,710)** | | |  |  |  | | | |
| Septicemia^a^ | 36,091 (33,993-38,190) | 33,883 (31,553-36,212) | 35,861 (33,375-38,346) | 35,674 (32,941-38,406) | 0.843 | | |  |
| Pneumonia^b^ | 38,467 (31,553-36,212) | 48,809 (44,649-52,969) | 38,096 (35,369-40,823) | 39,584 (36,334-42,834) | 0.202 | | |  |
| Unspecified^c^ | 15,154 (44,649-52,969) | 18,112 (16,590-19,634) | 14,754 (14,327-15,181) | 15,544 (14,267-16,822) | 0.001 | | |  |
| **Overall in 2012 (n =647,545)** | | |  | |  | | | |
| Septicemia^a^ | 34,429 (33,245-35,614) | 35,242 (33,676-36,807) | 33,149 (31,851-34,447) | 34,205 (32,516-35,895) | 0.218 | | |  |
| Pneumonia^b^ | 40,222 (38,508-41,935) | 55,839 (53,070-58,608) | 39,158 (37,256-41,060) | 41,143 (38,972-43,313) | 0.085 | | |  |
| Unspecified^c^ | 15,748 (15,308-16,188) | 18,832 (18,026-19,637) | 14,613 (14,190-15,036) | 15,520 (14,887-16,153) | <0.001 | | |  |
| **Overall in 2013 (n = 632,170)** | | |  |  |  | | | |
| Septicemia^a^ | 34,715 (33,305-36,125) | 34,698 (33,362-36,034) | 33,376 (31,821-34,931) | 33,745 (32,234-35,256) | 0.625 | | |  |
| Pneumonia^b^ | 39,881 (38,252-41,511) | 52,368 (49,663-55,073) | 38,828 (37,004-40,652) | 40,171 (37,938-42,403) | 0.221 | | |  |
| Unspecified^c^ | 15,601 (15,179-16,024) | 19,112 (18,304-19,920) | 14,481 (14,063-14,899) | 15,580 (14,949-16,211) | <0.001 | | |  |
| Note. MSSA = methicillin-susceptible *Staphylococcus aureus*; MRSA = methicillin-resistant *S. aureus*.  ^a^ ICD-9-CM: 038.11 and 038.12.  ^b^ ICD-9-CM: 482.41 and 482.42.  ^c^ ICD-9-CM: 041.11 and 041.12.  ^d^ Mean total cost (95% confidence interval) is presented in 2014 US$ to adjust for inflation | | | | | | | |  |

| **Supplementary Table 4. Crude and propensity-adjusted difference in costs between MRSA- and MSSA-related hospitalizations among patients with SSTIs and non-SSTIs, 2014** | | | | | |
| --- | --- | --- | --- | --- | --- |
|  | Crude Costs ($)^c^ | | Propensity-Adjusted Costs ($)^c^ | | |
|  | MRSA | MSSA | MRSA | MSSA | *p*-value |
| **Unspecified (n = 430,950)** | | |  |  |  |
| SSTI^a^ | 11,662 (11,362-11,962) | 13,089 (12,726-13,452) | 10,873 (10,597-11,148) | 11,098 (10,809-11,387) | 0.168 |
| Non-SSTI^b^ | 20,790 (20,099-21,480) | 24,680 (23,484-25,876) | 19,407 (18,731-20,083) | 20,548 (19,681-21,415) | 0.002 |
| Note. LOS = length of stay; MSSA = methicillin-susceptible *Staphylococcus aureus*; MRSA = methicillin-resistant *S. aureus*; SSTI = skin and soft-tissue infection.  ^a^ N = 221,600; ICD-9-CM: 680.xx (carbuncle and furuncle), 681.xx (cellulitis and abscess of finger and toe), 684 (impetigo), 682.xx (other cellulitis and abscess), 686.xx (other local infections of skin and subcutaneous tissue, 611.0 (inflammatory disease of breast), 704.8 (other specified diseases of hair and hair follicles, and 035 (erysipelas).  ^b^ N = 209,350; ICD-9-CM: 041.11 and 041.12, excluding SSTIs.  ^c^ Mean total cost (95% confidence interval). | | | | | |
